# Investigating fairness in global supply chains: applying an extension of the living wage to the Western European clothing supply chain

**DOI:** 10.1007/s11367-017-1390-z

**Published:** 2017-08-30

**Authors:** Simon Mair, Angela Druckman, Tim Jackson

**Affiliations:** Centre for Environment and Sustainability, University of Surrey, Guildford, UK

**Keywords:** Clothing, Fair wage, Fairness, Fashion, Input-output analysis, Living wage, Social impact, Social LCA

## Abstract

**Purpose:**

This paper explores the issue of fairness in global supply chains. Taking the Western European clothing supply chain as a case study, we demonstrate how applying a normative indicator in Social Life Cycle Assessment (SLCA) can contribute academic and practical insights into debates on fairness. To do so, we develop a new indicator that addresses some of the limitations of the living wage for SLCA.

**Methods:**

We extend the standard form of living wage available for developing countries to include income tax and social security contributions. We call this extension ‘living labour compensation’. Using publically available data, we estimate net living wages, gross living wages, and living labour compensation rates for Brazil, Russia, India, and China (BRIC) in 2005. We then integrate living labour compensation rates into an input-output framework, which we use to compare living labour compensation and actual labour compensation in the BRIC countries in the Western European clothing supply chain in 2005.

**Results and discussion:**

We find that in 2005, actual labour compensation in the Western European clothing supply chain was around half of the *living* labour compensation level, with the greatest difference being in the Agricultural sector. Therefore, we argue that BRIC pay in the Western European clothing supply chain was unfair. Furthermore, our living labour compensation estimates for BRIC in 2005 are ~ 35% higher than standard living wage estimates. Indeed, adding income taxes and employee social security contributions alone increases the living wage by ~ 10%. Consequently, we argue there is a risk that investigations based on living wages are not using a representative measure of fairness from the employee’s perspective and are substantially underestimating the cost of living wages from an employer’s perspective. Finally, we discuss implications for retailers and living wage advocacy groups.

**Conclusions:**

Living labour compensation extends the living wage, maintaining its strengths and addressing key weaknesses. It can be estimated for multiple countries from publically available data and can be applied in an input-output framework. Therefore, it is able to provide a normative assessment of fairness in complex global supply chains. Applying it to the Western European clothing supply chain, we were able to show that pay for workers in Brazil, Russia, India, and China is unfair, and draw substantive conclusions for practice.

**Electronic supplementary material:**

The online version of this article (10.1007/s11367-017-1390-z) contains supplementary material, which is available to authorized users.

## Introduction

The idea of ‘fairness’, and in particular fair wages, is a central issue for Social Life Cycle Assessment (SLCA) (Wang et al. 2016; Croes and Vermeulen 2016a; Croes and Vermeulen 2016b). Both SLCA and associated life cycle thinking methods (such as socially extended input-output analysis, e.g. Alsamawi et al. 2014b) are able to bring new dimensions to debates on fairness, particularly through exploration of fairness in global supply chains. However, fairness is a contested concept—it is understood differently by people who hold different worldviews. Quantified indicators can clarify how a contested concept is being operationalized in a particular application. However, to do this, they must be explicitly derived from a particular worldview (Mair et al. 2017). In the context of fairness, this means building an indicator from normative principles that describe what constitutes fairness in the view of the analyst. Such an indicator can provide clarity and depth to explorations of fairness, but has rarely been applied to date. Therefore, in this study, we explore the issue of fair pay in global supply chains, taking the Western European clothing supply chain as a case study and applying a novel, normative, and robust indicator of fairness: living labour compensation.

### The challenge of measuring fairness

Much recent work has tackled the issue of fairness in global supply chains. Amongst other things, this work has documented large differences in the wages paid within global supply chains: workers in developing and emerging economies are paid much less than workers in more affluent countries. Alsamawi et al. (2014a) show that this is the case in most supply chains serving developed countries, while several other studies explore this in more detail in case study supply chains, most notably clothing supply chains. For example, Mair et al. (2016) find that in the Western European clothing supply chain, workers in Western Europe are paid 30 times more than workers in Brazil, Russia, India, and China (BRIC). Similarly, Zamani et al. (2016) and Roos et al. (2016) identify several parts of the Swedish clothing supply chain where workers are at risk of earning less than 2 US dollars (USD) per day.

But how much do such studies actually tell us about fairness? For many commentators, it is not clear that a wage is unfair just because it is low by international standards. Conversely, it is often noted that not only do many workers in developing countries freely enter into jobs that have very low pay by international standards, but jobs that provoke the loudest calls of unfairness (such as those in garment factories) are in high demand (Dicken 2011; Tokatli et al. 2011; Clark and Powell 2013).

The key issue here is that fairness is a subjective and contested concept, so without an explicitly normative measure of fairness, it is not clear what it means for a low wage in developing countries to be ‘unfair’. Contested concepts are common in SLCA, and analysts can seek to mitigate the effects of the subjectivity that they bring to the analysis by ensuring that the subjective elements have a broad base of support, preferably with a grounding in international treaties or agreements (UNEP 2009).

As a result, in the SLCA community, living wages are seen as providing a measure of wage fairness (Neugebauer et al. 2014; Croes and Vermeulen 2016b) and have played a key role in judging the ‘fairness’ of wages in several SLCA applications (e.g. Ekener-Petersen and Finnveden 2013; Umair et al. 2015; Roos et al. 2016; Traverso et al. 2016). The strength of the living wage is that it provides a widely accepted notion of a ‘fair’ wage based on four clear and normative principles (Glickman 1999; Labour Behind the Label 2015): (1) a wage that provides for a better than subsistence lifestyle; (2) allows a worker to support their family; (3) is earned within a standard working week and does not rely on overtime; (4) and allows for financial security. Therefore, living wages provide a widely agreed upon, and normative, benchmark: wages that do not meet these four criteria are deemed ‘unfair’.

### Problems with living wages

There are two types of investigations into the fairness of pay in supply chains, of which the first is to assess the fairness of worker payments as things currently stand. To date, this has been the focus of most applications of living wages in SLCA (e.g. Hosseinijou et al. 2014). The second is to assess how we might make changes in order to make things fairer. Currently, this is the preserve of labour economists (e.g. Pollin et al. 2004), but as SLCA matures, it seems likely that recommendations for practice will begin to touch on such issues, and (as we will see) the life cycle approach means that such analyses are likely to raise new insights for practice.

However, the living wage has several limitations that make application along supply chains in either of these areas difficult. Firstly, using the living wage to assess current fairness requires us to know the wages of workers at every stage of the product life cycle. This is problematic, because wage data are difficult to obtain, especially in a suitably detailed form. The International Labour Organisation (ILO), for instance, provide wage data by country for most countries, but their occupational breakdown is at best highly aggregated and often non-existent (ILO 2015). This can be circumvented if site-specific analyses are carried out, but this is often infeasible in highly fragmented and complex global supply chains (Jørgensen et al. 2009; Zamani et al. 2016).

Moreover, many living wage estimates for developing countries do not account for income taxes, or employee social security contributions (Anker 2011a), while no living wage estimates account for employer social security contributions.^1^ This is likely to be problematic, because income and payroll taxes can be sizeable. As a result, a wage that looks like a living wage *before* tax and social security contributions may not provide enough income to live a decent life *after* tax and social security contributions (Anker 2005). Moreover, income tax and social security regimes vary widely between countries, potentially creating differences in national living wage rates (Anker 2005). Therefore, countries with net living wages of comparable sizes may have very different gross living wages, and analysts must be careful to ensure that they are comparing like with like if they are to realistically assess fairness.

Finally, because living wages do not include employer social security contributions, they underestimate the cost of living wages from the employer’s perspective and are therefore an unsuitable basis for making practical recommendations on how to improve supply chain fairness. Because employer social security rates vary widely across countries, the cost of a living wage could vary substantially across countries, even if the living wage itself is the same across countries. This may have practical implications for both transnational firms (in choosing where to locate their supply chain operations) and organisations lobbying for a common wage floor across countries (Asia Floor Wage 2014).

To address these limitations, in this paper, we extend living wages to include social security contributions and tax allowances. In effect, we estimate the level of labour compensation required to support a decent standard of living. The resulting measure, living labour compensation, is therefore an extension of the living wage, maintaining its normative principles but addressing the key weaknesses set out above. Because living labour compensation includes taxes and employee and employer social security contributions, it is comparable to labour compensation as defined in the System of National Accounts (European Comission et al. 2008). An additional advantage is that labour compensation includes estimates of the financial value of in-kind payments, so using it as the comparator mitigates the risk of inflated differences between living wages and wages caused by payments-in-kind. Finally, labour compensation data are readily available in input-output tables, meaning that living labour compensation can be readily applied in input-output-based SLCA.

### Exploring fairness in the clothing supply chain

In this paper, we address the issue of wage fairness specifically in the context of the Western European clothing supply chain. In clothing production, wages and fairness have been contentious issues for many years (e.g. Rivoli 2006), and, as noted above, several recent SLCA explorations of fairness have focused on the clothing supply chain (Mair et al. 2016; Roos et al. 2016; Zamani et al. 2016). By focusing on the Western European clothing supply chain, we aim to (1) demonstrate the usefulness of a normative standard of fairness in the form of living labour compensation and (2) make recommendations to both practitioners working to make the Western European clothing supply chain fairer and researchers working in the area of fairness more generally.

The rest of this paper is structured as follows. In section 2, we develop the living labour compensation indicator and describe its integration into an input-output model. In section 3, we present living labour compensation estimates for the BRIC countries and show the gap between BRIC living labour compensation and observed BRIC labour compensation in the Western European clothing supply chain (both for 2005). In section 4, we discuss the implications of our results for research and policy. Section 5 concludes.

## Methods

**Fig. 1:**
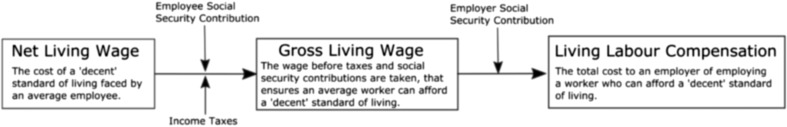
Relationship between net living wages, gross living wages, and living labour compensation

In this section, we extend the living wage to living labour compensation, using publically available data for BRIC. The BRIC countries were chosen, because they are all thought to be important in the clothing supply chain (Allwood et al. 2006; Pickles 2012; Mair et al. 2016). This is particularly the case for the year 2005, which we take as our case study year. Moreover, since the term was coined by O’Neil (2001), the BRIC countries have been extensively analysed as a single economic unit. It is also worth noting that there are little reliable data available for many of the other Asian countries involved in the Western European clothing supply chain. This is especially true of input-output data. In section 2.1, we outline a standard procedure for estimating internationally comparable *net* living wages (living wages that do not account for taxes). We then build on this, describing our method for incorporating income taxes and employee social security contributions to arrive at *gross* living wages. Finally, we demonstrate how to add in employer social security contributions to arrive at *living labour compensation*. Figure 1 illustrates the relationship between the three concepts. For each of the BRIC countries, employee and employer social security data is taken from Social Security Association (SSA 2015) (for the year 2005), and income tax data from Ernst and Young (2006) (for the year 2006).^2^

### Estimating net living wages

Our estimates of net living wage rates for the BRIC countries are based on the method developed by Anker (2005, 2006a, 2006b) and are methodologically similar to several other estimates such as the Asia Floor Wage (Merk 2009). We choose this approach because it has become a benchmark for living wage discussions both in the academic literature and by activists (e.g.Vaughan-Whitehead 2010; Action Aid 2011; Roos et al. 2016). As these methods are well described elsewhere (Anker 2006a, 2006b, 2005), we only provide an overview here. Full details of our calculations are given in Appendix A (Electronic Supplementary Material). All data are from public sources, cited in the text below.

First, for each BRIC country, we specify and cost a nutritionally sound diet incorporating country level preferences for food types. The model diets were constructed using country-specific data on food preferences (from FAOSTAT 2015) and consumer food prices (from ILO 2015). The ILO database provides the price *paid by consumers* for 93 food commodities, allowing relatively detailed pricing. The most up-to-date food price data in the ILO database were for 2000. These were converted to 2005 prices using food-specific consumer price indices (CPI; also from ILO 2015). Our model diet assumes 2100 Kcal is sufficient for a good but basic standard of living (Bassett and Winter-Nelson 2010; Economic Research Service and USDA 2012; World Food Programme 2015). However, to ensure our model diet meets nutritional needs beyond daily calorie intake, we follow guidelines from the World Health Organisation and the Food and Agricultural Organisation (WHO and FAO 2003). For example, our model diets specify five 80 g portions of fruit and vegetables per day.

We then use Engel coefficients to estimate total living costs based on the model diets. Engel coefficients represent the average share of the total income spent on food. This approach is well established; see Anker (2011a) for more details. We use Anker’s (2005) Engel coefficients, as they are specific to low-income households and vary by development level. Multiplying food costs by the Engel coefficient gives us an estimate of the cost of a decent lifestyle for an average person in the country of interest. We then multiply this value by a scalar to convert from an individual to a household value. To simplify interpretation of our living wage estimates, we follow Merk (2009) and Xu et al. (2015) in choosing one standard family size and structure—2 adults and 2 children with one full time worker. However, in moving from per person to household costs, there are economies of scale. Therefore, most estimates of national poverty lines use an adult equivalence scale to convert between the two. We use Anker’s (2005) equivalency scale which assumes all household members have the same calorific needs, but different non-food cost needs.

Finally, we apply a savings allowance of 10%. This allows for planning for the future and ensures the living wage allows a decent standard of living during times of financial crisis. Therefore, writing the daily cost of food as *f*, the Engel coefficient as *α*, the household scalar as *β*, and the savings allowance as *s*, the annual cost of a decent life (*w* ^*n*^) can be written as,

1 $$ {w}^n=365f{\alpha}^{-1}\beta \left(1+s\right) $$

### Estimating gross living wages: adding income taxes and employee social security contributions

Although we called the output of Eq. (1), the annual cost of a decent life, Anker (2006a) and Merk (2009) use similar procedures to estimate their living wages. We would argue that our interpretation of Eq. (1) is more appropriate, because workers are required to pay income taxes and make social security contributions out of their wages, neither of which is captured in Eq. (1). Additionally, as Anker (2005) notes, income taxes can constitute a substantial payment on the part of an employee, as can social security contributions. Therefore, we incorporate a tax allowance in Eq. (1).

The first step is to estimate the effective income tax rate for each country of interest. We did this for the four BRIC countries for 2005 using information on income tax regimes from Ernst and Young (2006). The effective income tax rate is country-specific, as it is dependent on the tax bands within each country, which tax band the living wage falls into and any deductible allowances—all of which vary by country. Table 1 shows the value of the effective income tax rate applied in each country. In Brazil, India, and China, the living wage falls below the minimum threshold for income tax, so the effective income tax rate is zero. However, in 2005, Russia had a flat tax rate, and the living wage would be taxed at 10%.

**Table 1:** Effective income tax rates and employee social security contribution rates used in Eqs. 1 to 3. Income tax data from Ernst and Young (2006) and social security data from SSA (2015); all social security data relates to the year 2005; income tax data relates to 2006

Country	Effective income tax rate	Employee social security contribution rate
Brazil	–	0.0765
Russia	0.10	–
India	–	0.1365
China	–	0.11

Employee social security contributions (for the year 2005) were taken from the SSA international research program (SSA 2015). These also vary by country: Russia is the only BRIC country not to require employees make a contribution to social security (the Russian system is entirely funded by employer contributions). For all of the BRIC countries, the SSA report employee social security contributions on a gross wage basis—however, for some countries, social security contributions are reported on a net basis, so those looking to extend our analysis beyond BRIC should take care to check this. Table 1 reports the employee social security contribution rates used in this study. As with income tax, most countries have a variety of rates applicable at different wage levels. The figures in Table 1 are the effective rates for our living wage estimates, accounting for these thresholds.

Using the effective income tax rates, *γ*, and employee social security contributions rates, *δ*, we can estimate a personal tax allowance, *h*, that ensures a post-tax wage, *w* ^*n*^, as estimated in Eq. (1),

2 $$ h=\frac{w^n\left(\gamma +\delta \right)}{\left(1-\left(\gamma +\delta \right)\right)} $$

And the annual living wage paid to an employee before any deductions for employee social security contributions or personal income taxes are made (i.e. the gross living wage *w* ^*g*^) is,

3 $$ {w}^g={w}^n+h $$

### Estimating living labour compensation: adding employer social security contributions

We take the employer social security contribution rates, ε, associated with our gross living wage estimates for each of the BRIC countries from the SSA (2015). These are then used to estimate ‘living labour compensation’, *w* ^*l*^,

4 $$ {w}^l=\left(1+\varepsilon \right){w}^g $$

The employer social security rates used in our empirical calculations for BRIC countries in 2005 are given in Table 2. All estimates in the table are country level, but there is likely to be substantial sub-national variation. Possible sources of this variation are differences in rates for smaller employers and varying levels of compliance. China also represents a special case within BRIC, as some components of social security are set by provinces rather than nationally. This leads to substantial variation across the country. In 2014, for example, employer contribution rates were 8% in Guangzhou and 22% in Shanghai (PWC 2014). The SSA (2015) provide guideline estimates for countries as a whole, and we use these while recognising that regionally there will be substantial variation around them. As with employee social security contributions, where applicable the tax rates in Table 2 account for different thresholds and marginal rates of tax.

**Table 2:** Employer social security contribution rates. Taken from SSA (2015) for the year 2005

Country	Employer social security contribution rate
Brazil	0.2000
Russia	0.2820
India	0.2236
China	0.1200

### Incorporating living labour compensation into an input-output model

To explore the fairness of wages in a supply chain, the living labour compensation estimates can be incorporated into an input-output model. Input-output models use detailed data on economic interactions between sectors to model supply chains and their attendant impacts, and they have been widely applied for environmental and social life cycle assessment (for example, Kondo and Nakamura 2004; Zamani et al. 2016). Here, we use input-output analysis in order to compare actual labour compensation and living labour compensation for BRIC worker’s in the Western European clothing supply chain.

To incorporate living labour compensation into an input-output model, we convert our estimates of annual living labour compensation for an average worker in each of the BRIC countries into estimates of the living labour compensation for each economic sector. The first step in this process is to estimate the living labour compensation rate: the per hour cost of labour, where the living wage is paid.

The living wage should be able to be earned by workers in a normal working week—workers should not have to rely on overtime (Anker 2011a). Therefore, we divide the annual living labour compensation estimates by full-time working hours. We assume that full time means 48 h a week, 50 weeks a year. This is approximately in line with working time statutes in the BRIC countries (ILO 2011) and reflects a general international consensus that more than 48 h constitutes excessive working time (Lee et al. 2007).

We then multiply this living labour compensation rate by the actual number of hours worked in each economic sector to get an estimate of the total living labour compensation by sector. Estimates of hours worked for 35 industrial sectors in 41 countries are available from the World Input-Output Database^3^ (Dietzenbacher et al. 2013). By multiplying estimates of the hours worked in each sector by the living labour compensation rate, we obtain estimates of the cost of labour by sector *if* the average wage had been equal to the living wage.

Finally, we construct our living labour satellite account. For the purposes of this analysis, we are interested in seeing how existing labour compensation compares to fair labour compensation and exploring the implications of this for existing living wage research, civil society, and business initiatives. Most of these initiatives aim to bring all workers up to at least a living wage. Therefore, for our purposes, it is most appropriate to incorporate the living labour compensation indicator into our input-output model in a way that best reflects the scenario where (1) those sectors that currently pay labour compensation at a level lower than living labour compensation have their compensation raised to the living labour compensation level, while (2) those sectors already paying more than the living labour compensation level continue to pay this amount (in other words, no sector sees a reduction in their labour compensation).

Therefore, we compare our living labour compensation estimate for each sector against the World Input-Output Database (WIOD) labour compensation estimates by sector. For those sectors where our living labour compensation estimate is less than WIOD’s labour compensation value, we use the latter in our living labour satellite account. Therefore, subsequent results reflect the additional cost of paying the living labour compensation rate assuming that workers in those sectors already paid more than the living labour compensation remain at the same level of compensation. Our living labour satellite account can be found in Appendix B (Electronic Supplementary Material).

### Using living labour compensation to assess fairness in the Western European clothing supply chain

The living labour satellite account can be used in conjunction with an input-output model to estimate where in supply chains BRIC wages are lower than the living wage and are therefore unfair. To demonstrate this, we incorporate the living labour satellite account into the input-output model used by Mair et al. (2016) to investigate the Western European clothing supply chain. Therefore, the model is the same as that applied by Mair et al. (2016) but applying the living labour satellite account (**w** ^∗^):

5 $$ {\mathbf{E}}^{\ast}=\widehat{{\mathbf{w}}^{\ast}}\mathbf{LY} $$

where, * indicates estimates based on living labour compensation, ˆ indicates diagonalisation, **L** is the Leontief inverse describing the interactions between different economic sectors, and **Y** is the Western European household demand for clothing goods (based on the Classification of Individual Consumption According to Purpose clothing and footwear category) in 2005 at basic prices, and **E** ^∗^ is the cost of labour in the Western European clothing supply chain assuming a living wage was paid. For comparative purposes, we also estimate the cost of BRIC labour in the Western European clothing supply chain in 2005 (**E**) using the original WIOD labour compensation satellite account (**w**):

6 $$ \mathbf{E}=\widehat{\mathbf{w}}\mathbf{LY} $$

## Results

This section reports our estimates of living labour compensation in the BRIC countries in 2005 and how labour costs would have changed in the BRIC parts of the 2005 Western European clothing supply chain had a living wage been paid. In the interests of brevity, we do not attempt to validate our living wage results here. However, Appendix C (Electronic Supplementary Material) shows how our net and gross living wage estimates compare to other estimates available in the literature and discusses how they fare when compared to theoretical expectations. In general, our estimates of living wages compare favourably to those available elsewhere in the literature.

### Living wage versus living labour compensation estimates for BRIC 2005

Table 3 compares the living labour compensation estimates on a per worker basis for Brazil, Russia, India, and China in 2005 valued in USD at Market Exchange Rates (MER).^4^ In all cases, including taxes and social security contributions substantially increases gross living wage and living labour compensation estimates relative to the net living wage estimate. In fact, averaged across the four BRIC countries, the gross living wage is ~ 10% higher than the net living wage, and living labour compensation is ~ 35% higher than the net living wage. This average hides a small amount of variation between countries. Personal taxes and employee social security contributions are equal to an 8% increase in the net living wage in Brazil, 11% in Russia, 16% in India, and 12% in China. Employer social security contributions are 17, 22, 18, and 11% of the total living labour cost in Brazil, Russia, India, and China, respectively. Consequently, the increase from a net living wage to living labour compensation varies between 26% (in China) and 43% (in Russia—India comes close to Russia with a 42% increase, and Brazil sits in the middle with a 30% increase).

**Table 3:** Components of living labour compensation estimates for a single worker in each BRIC country in 2005, valued at current price USD MER. Numbers may not sum due to rounding

	Net living wage	*Income Tax*	*Employee social security*	Gross living wage	*Employer social security*	Living labour compensation
Brazil	2763	–	*229*	2992	*598*	3590
Russia	1936	*216*	–	2152	*607*	2760
India	1289	–	*204*	1493	*334*	1826
China	1902	–	*235*	2137	*256*	2394

Italics signify components added in the move from one living wage concept to another

It is also worth noting that there are substantial differences in the cost of a living wage worker to a foreign firm depending on where that worker lives. Employing a living wage worker in Brazil costs a foreign firm around twice as much as employing a living wage worker in India, for example. However, including tax allowances and social security contributions does not change the *relative* cost of living wages between countries.

We comment more fully on the implications of these results for fairness in section 4. However, it is worth briefly exploring this here. The key implications of these results, in terms of fairness, are that (1) not including income taxes and social security contributions in estimates of fair pay leads to a systematic underestimation of the cost of a fair wage—both from the perspective of the employee and the employer. (2) Although accounting for social security and income taxes increases the absolute cost of paying employees fairly, it does not change the relative cost between the BRIC countries: it is still cheaper to employ a worker on a fair wage in India, than in Brazil, for example, when the relevant taxes are added to the fair wage estimate. As a result, paying fair compensation does not necessarily challenge the capitalist logic of moving capital between countries to chase lower wage bills. We discuss this in more detail in section 4.2.

### The additional cost of living wages in the BRIC clothing supply chain

**Fig. 2:**
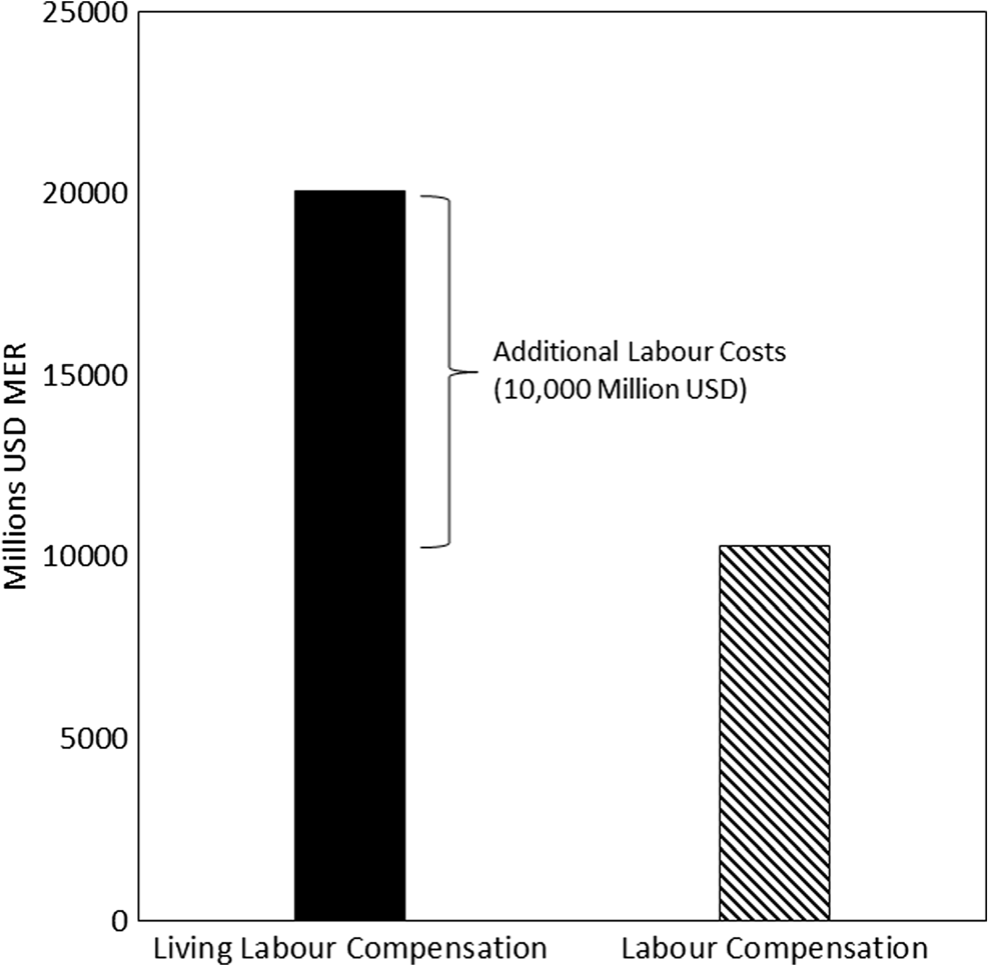
Change in the cost of labour in the BRIC parts of the Western European clothing supply chain associated with paying BRIC workers a living wage

Labour compensation for BRIC workers in the Western European clothing supply chain almost doubles when estimated using living labour compensation. The right hand bar in Fig. 1 shows that in 2005, the cost of BRIC labour in the Western European clothing supply chain was approximately 10 billion USD MER. The left hand bar in Fig. 2 shows that the cost of BRIC labour in the Western European, clothing supply chain assuming all workers were paid at least a living wage was approximately 20 billion USD MER. Put another way, BRIC workers in the Western European clothing supply chain are paid only half of a fair level of compensation, paid only half of what we estimate they need in order to be able to afford to live a decent life.

#### Sector level living labour premiums

**Fig. 3:**
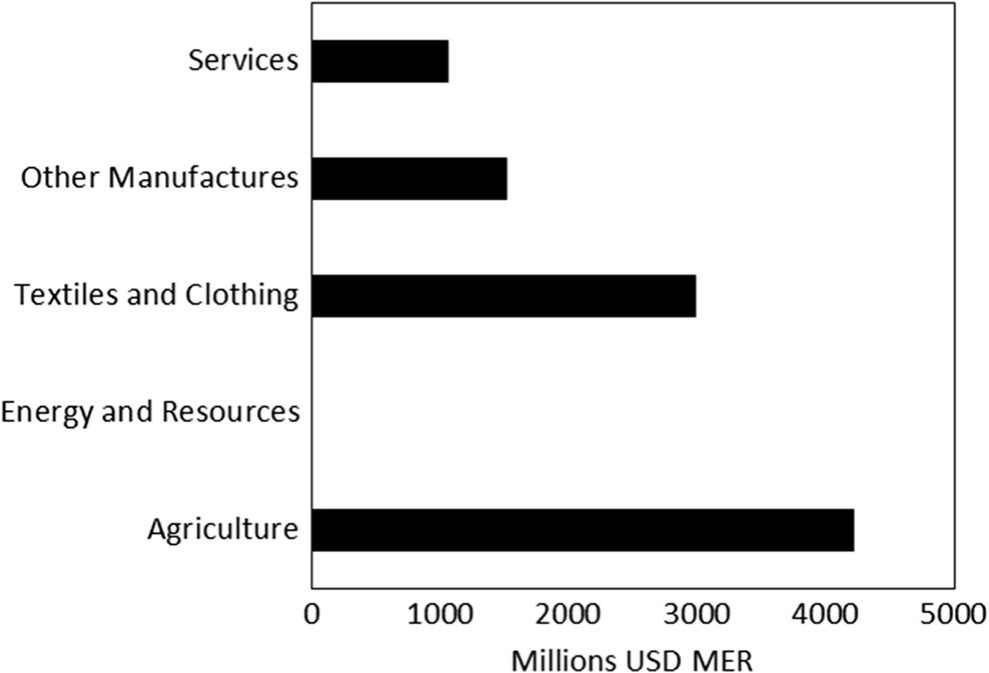
Absolute labour cost increase associated with the living wage in the BRIC countries by sector

**Fig. 4:**
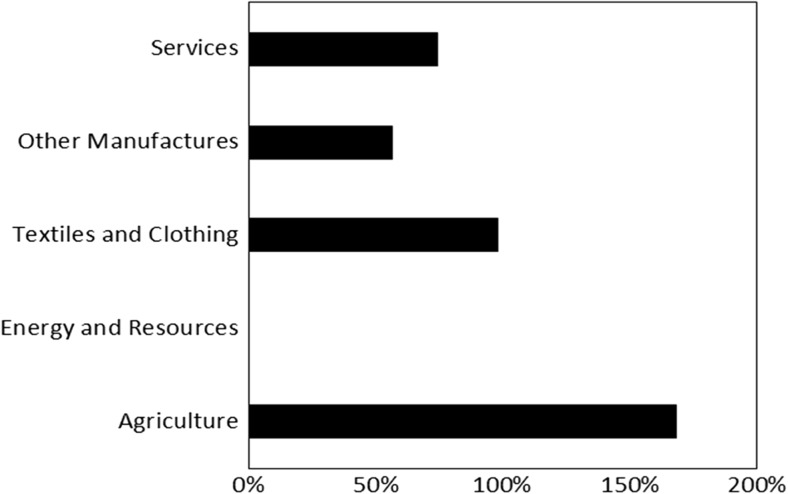
Relative labour cost increase associated with the living wage in the BRIC countries by sector

Figures 3 and 4 show how the additional cost to employers of fairly compensating BRIC workers in the Western European clothing supply chain would be distributed across sectors. As expected, in most sectors, worker compensation was below the living labour compensation level, so paying a fair compensation rate represents a cost increase.

In both absolute (Fig. 3) and relative (Fig. 4) terms, the biggest cost increase would be in the Agricultural sector where costs would increase by 4 billion USD MER (168%). This suggests that wages in the agricultural sector are the most unfair. The finding of a big gap between a fair compensation level and the actual compensation level is intuitive because of the low wages, poor working conditions, and low levels of labour productivity known to characterise many agricultural sectors in low-income countries, both within and beyond clothing supply chains. (e.g. Kalecki 1960/1993; Rivoli 2006; Mair et al. 2016). Likewise, the large cost increase in the textiles and clothing sector (3 billion USD MER, 98%) may reflect the perceived low skills of garment workers and the ease with which they are replaced (Li and Edwards 2008). However, these explanations do not justify the unfairness of this setup, which in the living wage/living labour compensation framework is based on the principle of the right to a decent quality of life.

The Other Manufactures and Service sectors have smaller labour cost increases than the Textiles and Clothing and Agricultural sectors because wages in the Other Manufacturers and Service sectors were closer to the living wage in 2005. Similarly, our results show no increase in labour costs in the Energy and Resources sector, because labour compensation in this sector was greater than our living labour compensation estimates. Although these sectors contain very heterogeneous activities in our classification system (Appendix D, Electronic Supplementary Material), these results are consistent with general expectations around the wage rates in different industries. For example, the Energy and Other Resources sector includes the Mining and Quarrying, and the Electricity, Gas, and Water supply sectors. Both of these sectors were above the industry average for the 2000–2005 ILO sectoral wage estimates for Brazil, Russia, and China (data for India was unavailable) (ILO 2015).

### Study limitations

This study has a number of limitations; we highlight the major ones here. First, construction of the living wage/living labour compensation estimates rely on a number of assumptions. For example, the core of the method is a mechanistic application of the Engel coefficient, which can be problematic (as discussed in Anker 2011b). Similarly (as discussed in section 2.3), we use national level estimates of income tax and social security even though these are known to vary by region and individual circumstance. As a result, our living wage and living labour compensation estimates should only be understood as averages that broadly reflect the cost of living across each of the BRIC countries.

The second major limitation is that our data are from 2005. But, while not ideal, we believe that using data from 2005 does not detract from the overall value of the study, as the fundamental characteristics of the system are unlikely to have changed since 2005. In 2005, the Western European clothing supply chain was dominated by the BRIC countries principally because the desire to keep costs low encouraged Western European retailers to source from low wage regions and to fragment the supply chain through sub-contracting (Mair et al. 2016). While the details of the supply chain will have changed since 2005, there is no reason to believe that the relative difference between the living labour compensation and actual labour compensation has been significantly altered by these changes. This is because the basic dynamic of the supply chain remains the same: retailers still choose to source in a way that reduces their costs and supply chains are still fragmented (Mair 2016). This is supported by the fact that studies using more recent (though less comprehensive) data suggest workers in clothing supply chains are still paid less than a living wage (e.g. McMullen et al. 2014).

Finally, our application of living labour compensation uses input-output analysis and is subject to all the usual limitations of this approach. The limitations of input-output analysis are well documented elsewhere; therefore, we will only briefly outline the key issues here. Interested readers are directed to Miller and Blair (2009), for comprehensive coverage. Firstly, input-output analysis is a linear model, meaning that the model does not account for returns to scale either in economic or social terms. Secondly, input-output analysis assumes that each sector produces a single homogenous output, whereas, in reality, sectors produce multiple outputs. For example, the agricultural sector produces both cotton and beef, which have very different production systems. Finally, the multi-regional form of input-output analysis used in this paper makes additional assumptions. This is because the kind of detailed trade data it requires is rarely available. Specifically, multi-regional input-output analysis usually assumes that intermediate and final consumption share the same proportion of imports (this is known as the import proportionality assumption). WIOD improves upon this by differentiating between intermediate and final imports using detailed Comtrade data (Dietzenbacher et al. 2013). However, this only allows WIOD to distinguish between intermediate and final demand. No such distinction is made within those categories.

## Discussion

### How fair is BRIC pay in the Western European clothing supply chain?

Our analysis provides a robust basis for arguing that BRIC pay in the Western European clothing supply chain is unfair. We found a substantial difference between living labour compensation (i.e. a fair compensation level) and observed labour compensation in the Western European clothing supply chain. Our results suggest that it would cost an additional 10 billion USD MER to reach a ‘fair’ level of pay for BRIC workers in the Western European clothing supply chain. This figure is equal to almost doubling the cost of this labour in BRIC in 2005. This result supports the argument that substantial inequities persist in affluent country apparel supply chains (e.g. Pollin et al. 2004; Mair et al. 2016) and supports the more general argument that many developing country workers in global supply chains are treated unfairly (Simas et al. 2014; Alsamawi et al. 2014a).

Likewise, our results suggest that workers in the Agricultural sector have the most unfair levels of labour compensation: we found that the biggest difference between a fair level of labour compensation and actual labour compensation was in the Agricultural sector. This result shows the need for the full supply chain, and in particular, the agricultural sector, to be considered by Western European retailers and brands if they are genuinely committed to fair supply chains. However, despite commitments to full value chain assessment in some quarters (e.g. Scherman 2015; New Look 2011; ETI 2015), there remains a major focus on garment factory workers in most discussions of social sustainability in the textile and clothing sector (e.g. Miller and Williams 2009; Labour Behind the Label 2015). There may be legitimate reasons for Western European retailers to exclude agricultural workers from living wage commitments. For example, it may be infeasible for Western European brands to dictate labour costs in the agricultural stages. However, it is important that these reasons are made explicit. Moreover, there is a risk that increasing wages for garment factory workers could squeeze wages further down the value chain if garment manufacturers attempted to absorb the additional costs of fair pay by pressuring their suppliers to provide them with cheaper materials.

### Implications for existing fair wage initiatives and research

Our results suggest that current research may underestimate the true cost of fair wages. We found that in BRIC, both living labour compensation and gross living wage estimates are substantially higher than net living wage estimates. This has two important implications for those working on issues of fair wages. First, our findings suggest that to an employer, social security contributions mean that the cost of paying a fair wage is substantially higher (in our case ~ 35%) than standard living wage estimates would suggest. This is a large additional increase for employers to pay, something that should be acknowledged in any recommendations to pay living wages. It is also likely to affect the ability of firms to pay fair wages, and this could be important in consequential SLCA. Secondly, when evaluating the fairness of compensation, SLCA practitioners should ensure that employee social security and income tax payments are accounted for in the living wage estimates they use. Otherwise, compensation that appears to allow workers to live a ‘decent life’ and therefore appears to be ‘fair’ may in fact be insufficient to allow access to a decent life and may be deeply unfair.

We also found considerable variation in living labour compensation across countries, despite fairly consistent tax and social security contribution rates. According to our estimates, employing a living wage worker in India costs around half as much as employing a living wage worker in Brazil. This is well established for net living wages in developing countries (Anker 2006a; Merk 2009), and we show that it persists once differing levels of taxes are accounted for. It is a reflection of the fact that living wages are inherently subjective and influenced by general living standards and levels of economic development. Consequently, those countries that are lower on the development ladder will have a lower living wage than countries more economically developed countries. This is why our estimate of a living wage for India is lower than our estimate of a living wage for China, for example.

This is important because variation in the cost of fair compensation between countries has been implicitly ignored in studies looking at the effects of paying fair wages in apparel supply chains. For example, Miller and Williams (2009) estimate how doubling the wages of garment factory workers in the Philippines producing a men’s knit shirt would affect prices. Similarly, Pollin et al. (2004) estimate how the price of a men’s shirt might be changed if Mexican garment factory workers were paid a living wage. Others make the same simplifying assumption of only examining workers in one country (Birnbaum 2000; WRC 2005). Given the fragmented nature of global value chains, the variation in both living wage rates and living labour compensation rates suggests that such studies may not be generalisable outside of their specific contexts.

Moreover, the cross-national variation in living labour compensation rates implies that brands and retailers in Western Europe could pay their employees fairly while continuing to chase the lowest global labour costs. The only difference from the current system would be that the lowest possible wage would be a fair wage. Therefore, if all retailers selling clothing goods in the Western Europe agreed to a living labour compensation floor, there is no reason to believe that capitalist competition based on wages would stop. Firms could still shift production from one country to another looking for the lowest possible fair wage. A positive take on this would be that globalisation in the textile and clothing industry supply chain could continue to function in the same development role as it has historically, providing employment to workers in the lowest income countries (e.g. Rivoli 2006; Tokatli et al. 2011).

However, there is also another perspective on this, namely that any ‘race to the bottom’ (even a bottom considered ‘fair’) is inherently undesirable. This is the position of campaigns like the Asia Floor Wage Alliance (Merk 2009), a group of unions and labour activists from across Asia whom advocate:

a regional collective bargaining strategy … [intended to]… to counter the threat of capital mobility …[and to]… prevent competition based on wage levels between Asian garment exporters and to make sure that gains are shared along the supply chain. (Merk 2009 P.39)

This regional collective bargaining strategy is based on a single net living wage estimate which is applied to Bangladesh, China, India, Indonesia, Sri Lanka, and Thailand. Although we have a slightly different geographical focus, our results do provide insights into the Asia Floor Wage Alliance approach.

As it stands, the Asia Floor Wage Alliance is likely to underestimate the true cost of fair compensation. The Asia Floor Wage Alliance fair pay estimate currently does not account for taxes in any form. Our results suggest that including personal income taxes in the Asia Floor Wage is probably unnecessary, as in most countries with progressive taxes living wages would fall within tax free allowances (though this is not certain and could change). However, we saw that employee and employer social security contributions can substantially increase the cost of fair labour. Consequently, the Asia Floor Wage may not currently be fair.

Moreover, the Asia Floor Wage may not stop competition based the cost of labour. This is because the Asia Floor Wage is a single figure applied to several countries but does not include social security contributions. This is an issue, because social security contribution rates vary between countries. When we move from a living wage to living labour compensation, the relative cost of a fair wage does not change between countries. But, this is because our living wage rate varies between the countries. If the same net living wage was adopted across several countries (as is the case with the Asia Floor Wage), the cost of labour could still change when employee and employer social security contributions were included. Put another way, the Asia Floor Wage does not fully account for the full costs of paying a living wage from an employers perspective. This is a problem, because it is the cost to employers that incentivises the shifting of capital between countries. If the single Asia Floor Wage were implemented across a range of countries, firms looking to minimise their labour bills might simply look to employ workers in countries with little or no social security provision. On this basis, our results lead us to believe that the Asia Floor Wage Alliance proposals would benefit from thorough investigation of these issues in their specific geographical context and incorporation of labour tax and social security estimates into their calculations.

## Conclusions

This paper argues that any assessment of fairness should be normative, and that any assessment based on the living wage concept must take into account personal income taxes and the social security payments made by employees and employers. Consequently, we have proposed living labour compensation as a new indicator for assessing wage fairness in global supply chains. Living labour compensation maintains the strengths of the living wage (namely a widely accepted notion of what constitutes a fair wage) but improves on the shortcomings of living wage indicators by incorporating the additional costs of social security contributions and taxes. Consequently, it is a better reflection of the true cost of living wages and is more in line with standard labour assessment techniques. Additionally, we have demonstrated that the indicator can be compiled for multiple countries and applied in input-output analysis.

Applying the living labour compensation indicator to the BRIC countries in the Western European clothing supply chain showed that the labour compensation bill for workers in the BRIC countries would have almost doubled had a living wage been paid in the 2005 Western European clothing supply chain. This provides a robust basis to the claim that wages were unfair. Taking a full supply chain approach highlighted the low pay of agricultural workers—a group often neglected in discussion of social sustainability in the textiles and clothing context. We also highlighted the fact that including taxes and social security contributions substantially changes the cost of living wages, particularly from an employer’s perspective.

On the basis of these findings, we were able to make several substantive and specific recommendations for researchers, activists, and companies working on labour fairness issues in global supply chains. Although the focus of our application here was the Western European clothing supply chain, our results have broader implications. For example, we highlighted the need to consider multiple countries in living wage research and demonstrated how this could be done using living labour compensation in an input-output framework. Likewise, we argued that using living labour compensation strengthens the arguments about unfairness in clothing supply chains and suggested that by ignoring key elements of worker compensation, charities, and activist groups may undermine their own positions. Although such issues receive large amounts of attention in the context of clothing supply chains, they are common to most supply chains serving affluent countries. Consequently, the investigation of issues of fairness in global supply chains using the normative indicator approach outlined in this paper has implications for researchers and practitioners in multiple contexts.

## Electronic supplementary material

ESM 1 (XLSX 42 kb)

ESM 2 (XLSX 68 kb)

ESM 3 (DOCX 432 kb)

ESM 4 (DOCX 25 kb)

## Footnotes

1 The only living wage work that accounts for employer social security contributions is a study into Chinese wages by Xu et al. (2015). They add a 30% premium to the Chinese living wage, but do not use this in their analysis.

2 We use Ernst and Young 2006, as this is the data going furthest back in time that we could find on income tax regimes. At the time of writing, information on personal tax regimes around the world between 2006 and 2014 can be found at http://www.ey.com/GL/en/Services/Tax/Global-tax-guide-archive.

3 We use WIOD primarily, because it is freely available and constructed from publically available data. The process should work with other global input-output databases. See Dietzenbacher et al. (2013) for more on WIOD.

4 We use USD MER here as firms pay wages and count costs at market valuations.

## References

[CR1] Action Aid (2011) Eight steps towards a living wage: a costing model for clothing brands and retailers Available at: https://www.actionaid.org.uk/sites/default/files/doc_lib/actionaid_living_wage_model_-_final.pdf (Accessed: 17/11/2015)

[CR2] Allwood JM, Laursen SE, Rodriguez CM, Brocken NMP (2006) Well dressed? The present and future sustainability of clothing and textiles in the United Kingdom. University of Cambridge Institute for Manufacturing, Cambridge

[CR3] Alsamawi A, Murray J, Lenzen M (2014). The employment footprints of nations. J Ind Ecol.

[CR4] Alsamawi A, Murray J, Lenzen M, Moran D, Kanemoto K (2014b) The inequality footprints of nations: a novel approach to quantitative accounting of income inequality. PLoS ONE 9:(10) 10.1371/journal.pone.0110881 PMC4212986 25353333

[CR5] Anker R (2005) A new methodology for estimating internationally comparable poverty lines and living wage rates’, ILO Working Paper 72

[CR6] Anker R (2006). Living wages around the world: a new methodology and internationally comparable estimates. Int Labour Rev.

[CR7] Anker R (2006). Poverty lines around the world: a new methodology and internationally comparable estimates. Int Labour Rev.

[CR8] Anker R (2011a) Estimating a living wage: a methodological review. Conditions of Work and Employment Series No. 29 ILO

[CR9] Anker R (2011b) Engel’s law around the world 150 years later, PERI Working Paper No. 247. Available at: http://core.ac.uk/download/pdf/6307249.pdf (Accessed: 09/11/2015)

[CR10] Asia Floor Wage (2014) What is the Asia floor wage. Available at: http://www.cleanclothes.org/livingwage/what-is-the-asia-floor-wage (Accessed: 09/12/2014]

[CR11] Bassett TJ, Winter-Nelson A (2010) The atlas of world hunger. University of Chicago Press

[CR12] Birnbaum D (2000). Birnbaum’s guide to winning the great garment war.

[CR13] Clark J, Powell B (2013). Sweatshop working conditions and employee welfare: say it ain’t sew. Comp Econ Stud.

[CR14] Croes P, Vermeulen W (2016). In search of income reference points for SLCA using a country level sustainability benchmark (part 1): fair inequality. A contribution to the Oiconomy project. Int J Life Cycle Assess.

[CR15] Croes P, Vermeulen W (2016). In search of income reference points for SLCA using a country level sustainability benchmark (part 2): fair minimum. A contribution to the Oiconomy project. Int J Life Cycle Assess.

[CR16] Dicken P (2011) Fabric-ating fashion: the clothing industries. In: Dicken P (ed) Global Shift: Mapping the Changing Contours of the World Economy. 6th ed: Sage Publications, pp 302-330

[CR17] Dietzenbacher E, Los B, Stehrer R, Timmer M, de Vries G (2013). The construction of world input–output tables in the WIOD project. Econ Systems Res.

[CR18] Economic Research Service and USDA (2012) (United States Department of Agriculture), International Food Security Assessment, 2012–22. Available at: http://www.ers.usda.gov/media/849266/gfa23.pdf (Accessed: 01/02/2015)

[CR19] Ekener-Petersen E, Finnveden G (2013). Potential hotspots identified by social LCA—part 1: a case study of a laptop computer. Int J Life Cycle Assess.

[CR20] Ernst and Young (2006) The Global Executive Available at: http://www.ey.com/Publication/vwLUAssets/Global_Executive_2006/$FILE/GE_2006_Global_Executive.pdf (Accessed: 10/05/2014)

[CR21] ETI (2015) Living Wages in Global Supply Chains: A new agenda for business Available at: http://www.ethicaltrade.org/sites/default/files/resources/living_wages_in_global_supply_chains.pdf (Accessed: 15/05/2015)

[CR22] European Comission, IMF, OECD, UN and World Bank (2008) System of National Accounts Available at: http://unstats.un.org/unsd/nationalaccount/docs/SNA2008.pdf (Accessed: 17/11/2015)

[CR23] FAOSTAT (2015) Food Balance Sheets Available at: http://faostat3.fao.org (Accessed**:** 02/01/2015)

[CR24] Glickman LB (1999) A living wage: American workers and the making of consumer society. Cornell University Press

[CR25] Hosseinijou S, Mansour S, Shirazi M (2014). Social life cycle assessment for material selection: a case study of building materials. Int J Life Cycle Assess.

[CR26] ILO (2011) Working Conditions Laws Database: ILO, Geneva Available at: http://www.ilo.org/dyn/travail (Accessed: 29/04/2016)

[CR27] ILO (2015) ILOSTAT Available at: http://www.ilo.org/ilostat/ (Accessed: 01/03/2015)

[CR28] Jørgensen A, Hauschild M, Jørgensen M, Wangel A (2009). Relevance and feasibility of social life cycle assessment from a company perspective. Int J Life Cycle Assess.

[CR29] Kalecki M, Osiatynski J (1960). Unemployment in underdeveloped countries. Collected works of Michal Kalecki.

[CR30] Kondo Y, Nakamura S (2004). Evaluating alternative life-cycle strategies for electrical appliances by the waste input-output model. Int J Life Cycle Assess.

[CR31] Labour Behind the Label (2015) Living Wage Available at: http://www.labourbehindthelabel.org/campaigns (Accessed: 15/02/2015)

[CR32] Lee S, McCann D, Messenger J (2007). Working time around the world: trends in working hours, laws and policies in a global comparative perspective.

[CR33] Li M, Edwards P (2008). Work and pay in small Chinese clothingfirms: a constrained negotiated order. Indus Rel J.

[CR34] Mair S (2016) Better rather than more? Exploring the sustainability implications of paying a living wage in the western European clothing supply Chain, PhD, University of Surrey

[CR35] Mair S, Druckman A, Jackson T (2016). Global inequities and emissions in Western European textile and clothing consumption. J Clean Prod.

[CR36] Mair S, Jones A, Ward J, Christie I, Druckman A, Lyon F (2017) A critical review of the role of indicators in implementing the sustainable development goals. In: Leal W (ed) Handbook of sustainability science and research. Springer

[CR37] McMullen A, Luginbühl C, Nolan K, Crabbé C, Ajaltouni N (2014) Tailored Wages Available at: http://www.labourbehindthelabel.org/campaigns/itemlist/category/294-report (Accessed: 13/02/2017)

[CR38] Merk J (2009) Stitching a decent wage across borders: the Asia floor wage proposal. Globalization & the Workplace 429. Available at: http://digitalcommons.ilr.cornell.edu/cgi/viewcontent.cgi?article=1423&context=globaldocs (Accessed: 01/02/2015)

[CR39] Miller D, Williams P (2009). What price a living wage? Implementation issues in the quest for decent wages in the global apparel sector. Global Soc Pol.

[CR40] Miller R, Blair D (2009). Input output analysis, foundations and extensions.

[CR41] Neugebauer S, Traverso M, Scheumann R, Chang YJ, Wolf K, Finkbeiner M (2014). Impact pathways to address social well-being and social justice in SLCA—fair wage and level of education. Sustainability.

[CR42] New Look (2011) New Look submission to 2011 update of Let’s Clean Up Fashion,. Available at: http://www.labourbehindthelabel.org/jobs/item/878

[CR43] O’Neill J (2001 ) Global EconomicsPaper No: 66, Building Better Global Economic BRICs: Goldman Sachs (Accessed**:** 11/04/2016)

[CR44] Pickles J (2012) Economic and social upgrading in apparel global value chains: public governance and trade policy. Available at: http://www.capturingthegains.org/pdf/ctg-wp-2012-13.pdf (Accessed: 19/04/2016)

[CR45] Pollin R, Burns J, Heintz J (2004). Global apparel production and sweatshop labour: can raising retail prices finance living wages?. Camb J Econ.

[CR46] PWC (2014) Social security systems around the globe Available at: http://www.pwc.com/gx/en/hr-management-services/pdf/social-security-country-profiles-march-2014.pdf (Accessed: 24/02/2015)

[CR47] Rivoli P (2006). The travels of a T-shirt in the global economy.

[CR48] Roos S, Zamani B, Sandin G, Peters G, Svanström M (2016). An LCA-based approach to guiding an industry sector towards sustainability: the case of the Swedish apparel sector. J Clean Prod.

[CR49] Scherman M (2015) "Sustainability is not about cost"—Anna Gedda Interview for Investment Perspectives on Share Radio, UK. Available at: https://audioboom.com/boos/3103086-sustainability-is-not-about-cost-anna-gedda-head-of-sustainability-hm#t=0m20s (Accessed: 21/04/2015)

[CR50] Simas M, Golsteijn L, Huijbregts M, Wood R, Hertwich E (2014). The “Bad Labor” footprint: quantifying the social impacts of globalization. Sustainability.

[CR51] SSA (2015) (Social Security Association), Office of International Programs Home Page,. Available at: http://www.ssa.gov/international/ (Accessed: 19/02/2015)

[CR52] Tokatli N, Kizilgun O, Cho J (2011). The clothing industry in Istanbul in the era of globalisation and fast fashion. Urban Stud.

[CR53] Traverso M, Bell L, Saling P, Fontes J (2016) Towards social life cycle assessment: a quantitative product social impact assessment. Int J Life Cycle Assess. 10.1007/s11367-016-1168-8

[CR54] Umair S, Björklund A, Petersen E (2015). Social impact assessment of informal recycling of electronic ICT waste in Pakistan using UNEP SETAC guidelines. Resour Conserv Recy.

[CR55] UNEP (2009). Guidelines for social life cycle assessment of products.

[CR56] Vaughan-Whitehead D (2010). Fair wages: strengthening corporate social responsibility.

[CR57] Wang S, Hsu C, Hu A (2016). An analytic framework for social life cycle impact assessment—part 1: methodology. Int J Life Cycle Assess.

[CR58] WHO and FAO (2003) Diet, nutrition and the prevention of chronic diseases. WHO Technical Report Series,. Available at: http://whqlibdoc.who.int/trs/WHO_TRS_916.pdf?ua=1 (Accessed: 01/02/2015 12768890

[CR59] World Food Programme (2015) What is Hunger? Available at: http://www.wfp.org/hunger/what-is (Accessed: 04/02/2015)

[CR60] WRC (2005) (Workers Rights Consortium) The Impact of Substantial Labor Cost Increases on Apparel Retail Prices. Globalization & the Workplace 219. Available at: http://digitalcommons.ilr.cornell.edu/cgi/viewcontent.cgi?article=1206&context=globaldocs (Accessed: 27/05/2015)

[CR61] Xu Z, Chen Y, Li M (2015). Are Chinese workers paid the correct wages? Measuring wage underpayment in the Chinese industrial sector, 2005–2010. Rev Radic Polit Econ.

[CR62] Zamani B, Sandin G, Svanström M, Peters G (2016) Hotspot identification in the clothing industry using social life cycle assessment—opportunities and challenges of input-output modelling. Int J Life Cycle Assess. 10.1007/s11367-016-1113-x

