# Supplementary material for: Investigating fairness in global supply chains: applying an extension of the living wage to the Western European clothing supply chain
Source: Int J Life Cycle Assess. 2017 Aug 30;23(9):1862–73. doi: 10.1007/s11367-017-1390-z (PMC6428394; doi:10.1007/s11367-017-1390-z)
Supplement: Supplementary file 3 — (DOCX 432 kb) [file 11367_2017_1390_MOESM3_ESM.docx]

**Appendix C.**

**C 1 Comparative Assessment of Net Living Wage Estimates**

Figures C-1 and C-2 plot published living wage estimates for China (Figure C-1) and India (Figure C-2) between 2000 and 2009 alongside our net living wage, gross living wage and living labour compensation estimates. All values are converted to 2010 USD, MER. Overall, our estimates appear to be quite reasonable, being within the range of published estimates. For China, our net and gross living wage estimates fall toward the lower end of the spread of estimates, though considerably above the lowest estimates and lying close to the estimates for 2005 and 2006 by Xu et al., (2015) (Figure C-1). Similarly, for India our estimate is reasonably central, though closer to the top half of the range of estimates (Figure C-2).


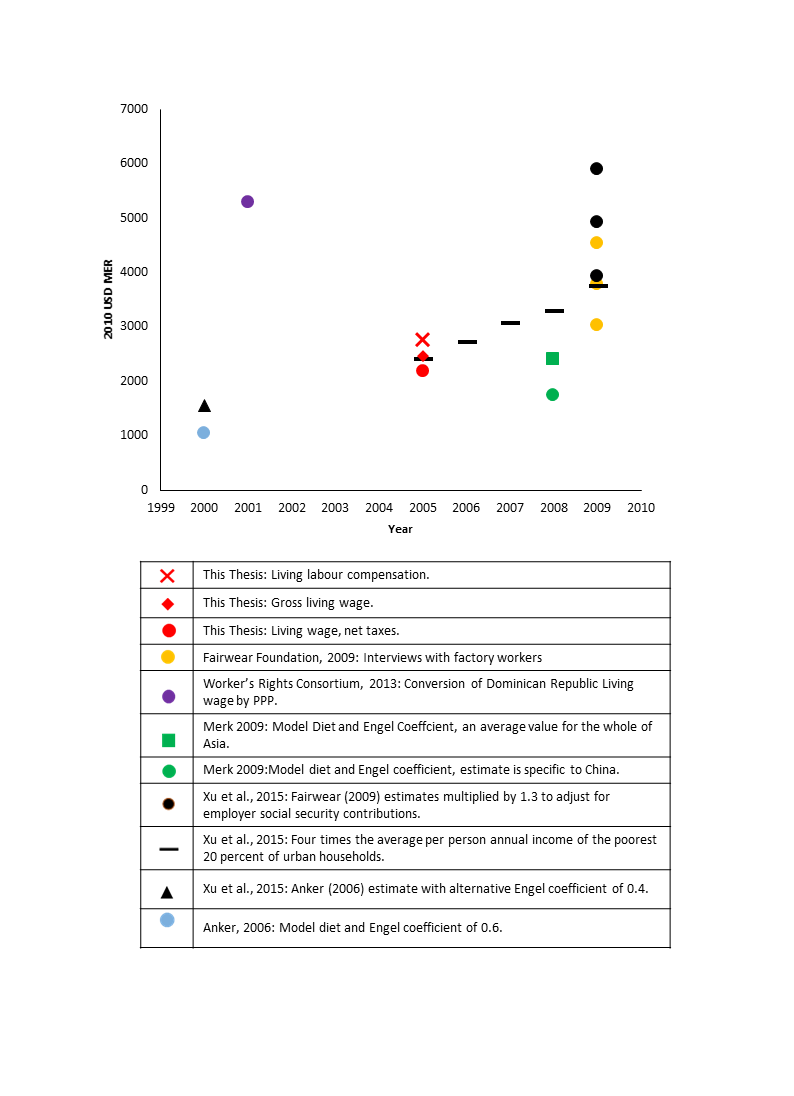


This study, living labour compensation

This study, gross living wage

This study, net living wage

Figure C‑1 Estimates of living wages in China 2000-2009. Markers of the same colour are from the same study; markers of the same colour but different shapes indicate same study but different method; markers of the same colour and shape indicate same study and same method but a different geographical focus


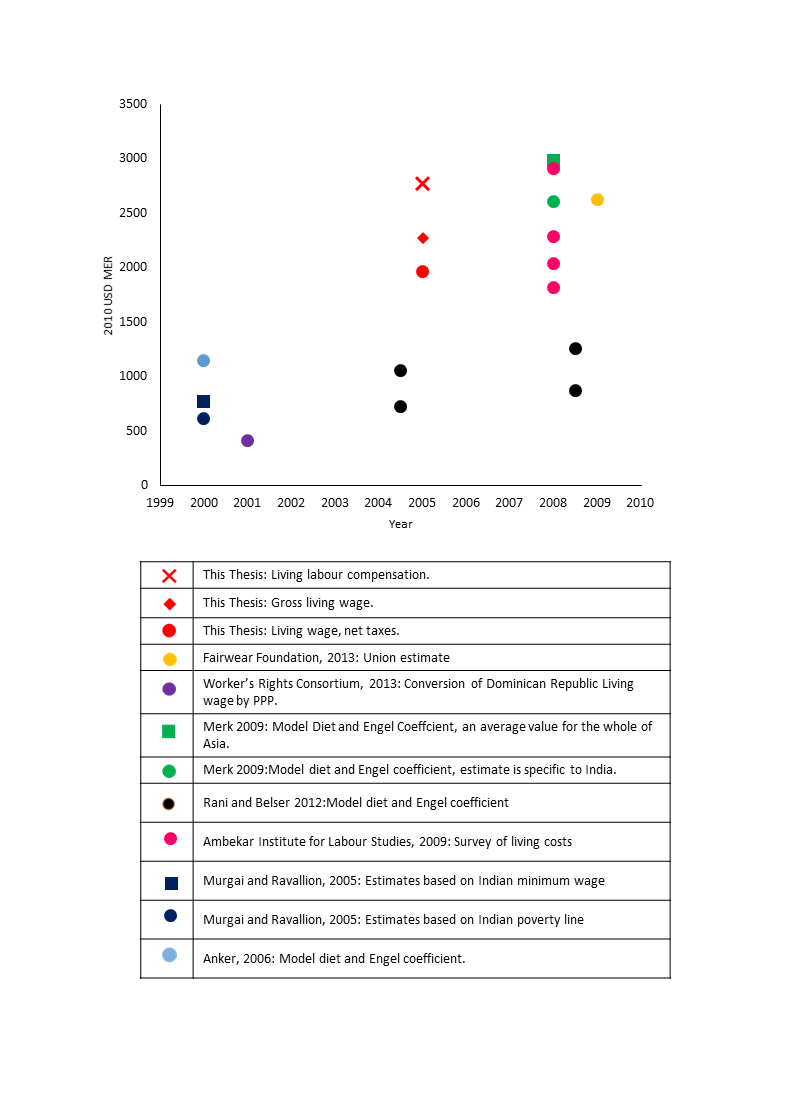


This study, net living wage

This study, living labour compensation

This study, gross living wage

Figure C‑2 Estimates of living wages in India 2000-2009. Markers of the same colour are from the same study; markers of the same colour but different shapes indicates same study but different method (see key); markers of the same colour and shape indicate same study and same method but a different geographical focus.

In Figures C-1 and C-2 the estimates methodologically closest to ours are Anker (2006) and Merk (2009), as both estimate food costs and apply an Engel coefficient. Neither Anker nor Merk account for taxes or social security, so the relevant comparison is with our net living wage. For both China and India, our net living wage estimate is substantially greater than Anker’s estimate. As we use the same Engel coefficient as Anker (0.6) and similar calorie requirements, the differences between our net living wage and Anker’s estimate must be due to differences in the specification and pricing of our model diets. We used the same food price database as Anker. However, we updated the prices using a food specific CPI. While we attempt to control for inflation in the living wage estimates in figures C-1 and C-2 using a general CPI, it is possible that this does not completely counteract our adjustment for inflation of food prices between 2000 and 2005. Additionally, Anker adjusts the price of rice downwards (by half in India and a third in China) on the grounds that cheaper forms of rice are available than those used in the ILO food prices database. This reduces the cost of his diets relative to ours. We do not do this in order to maintain the transparency and simplicity of our estimates. Regardless, as our living wage estimate is toward the lower end of the published estimates we do not think the differences between ours and Anker’s model diets are problematic.

We would expect Merk’s (2009) estimates for both China and India to be greater than our net living wage estimate as Merk uses a smaller Engel coefficient (Merk uses 0.5 for both China and India, while we use 0.6 and 0.7 respectively) and specifies a model diet with more calories (3000, to our 2100, see 4.2.2). In India this is the case, with Merk’s estimate being higher even than our gross living wage estimate. However, in China our net living wage estimate is greater than Merk’s. This could suggest that our Chinese model diet overstates the price of a good diet. However, it seems equally likely to us that the differences between our estimates, Anker’s and Merk’s highlight the limitations of mechanistic application of the Engel coefficient and not explicitly modelling the cost of other essential goods such as housing (Anker, 2011a, Anker, 2011b).

The only other living wage estimates that explicitly attempt to account for social security costs are the Xu et al., (2015) adjustments of the Fairwear Foundation (2009) estimates. Our living labour compensation estimate is considerably less than the social security adjusted Fairwear Foundation estimates. This is most likely because the Fairwear Foundation (2009) living wage estimates are greater than our gross living wage estimate. It is difficult to say why the Fairwear estimates are higher than our estimates as they are based on interviews which asked for desired earnings rather than budgets. This fact does, however, suggest that our estimates are conservative.

**C 2 Living Wages vs Average Wages in BRIC.**


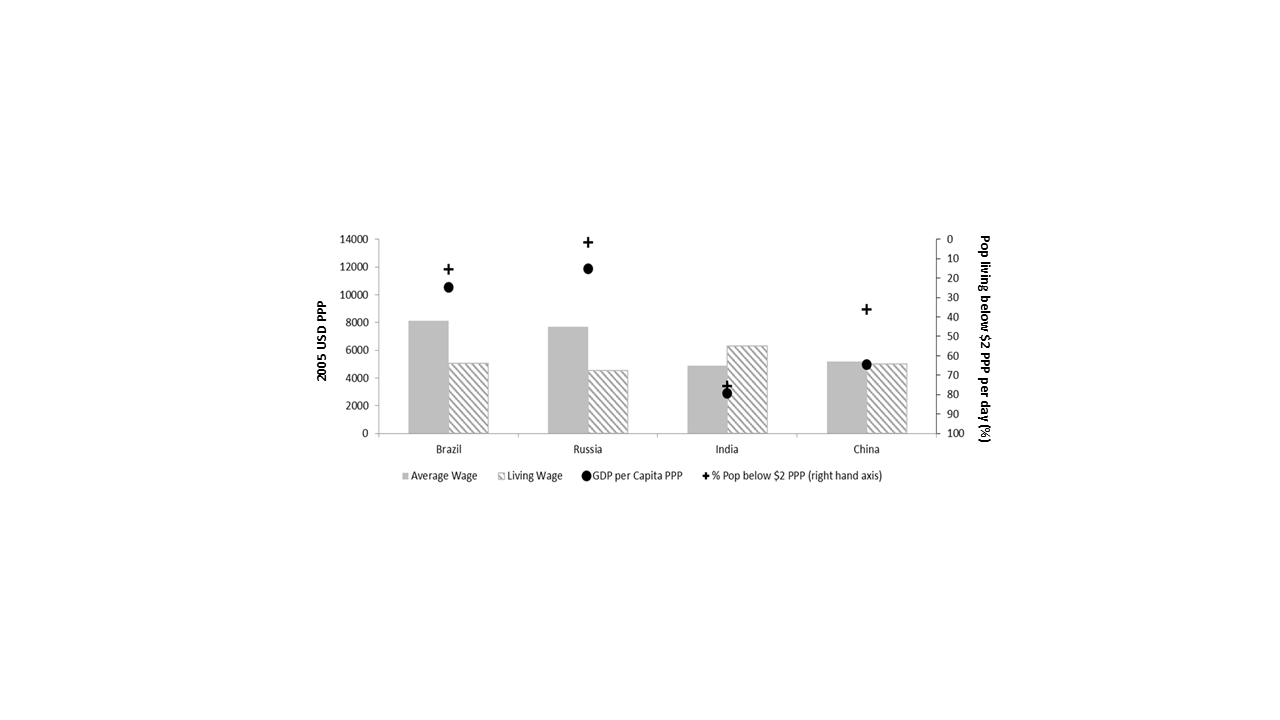
Figure C-3 shows our living wage estimates for BRIC in 2005 valued at USD PPP. Brazil and China have very similar PPP annual living wage values, both around 5000 USD PPP, and Russia is only ~500 USD PPP less. On the other hand, our estimate of India’s annual living wage much higher than all of our other estimates (around 1200 USD PPP higher than Brazil and China and 1700 USD PPP more than our Russian living wage estimate).

Figure 4‑3 Mean nominal annual earnings, living wage, GDP per capita and percentage of population living below the poverty line in BRIC countries in 2005.

That the Indian living wage is the highest in terms of USD PPP is a counter-intuitive result as it suggests that the cost of living is higher in India than in Brazil, Russia or China. In fact if PPP were a perfect measure of cross-national purchasing power discrepancy and our living wage estimates provided for the exact same standard of living in each country we would expect all the values to be the same in terms of USD PPP. Therefore (discounting the numerous problems with PPP estimation), the fact that Indian living wage estimate has a much higher value in USD PPP than those for Brazil, Russia and China suggests that our living wage estimates for India are too high, or the other estimates too small (which would also suggest problems with the ILO data or the Engel coefficients). In our judgement, the discrepancy is most likely the result of the Engel coefficient being too low and what look like surprisingly high food prices (the food prices listed for India in the ILO database comparable to Russian food prices at USD PPP – Appendix A). ILO food prices could be too high because a large number of food goods are being sold on the informal market, for example. This would be consistent with Figure C-2, which shows our estimate being on the high side of the published estimate for India.

That said, Figure C-3 also shows the ILO average earnings estimates, as well GDP per capita and the percentage of the population living on less than 2 USD PPP per day. We can see that Brazil and Russia have relatively high economic development and low poverty rates and, as we would expect, their average wages are above the living wage. Conversely, the gap between the living wage and the average wages is much smaller for China which has higher poverty rates and lower levels of economic development. In terms of income, India is the poorest country we look at, and has very low levels of economic development and extremely high levels of poverty. It makes sense, then, that our living estimate for India is higher than the average wage. Therefore, our estimates are broadly consistent with the theoretical expectations set out above.

**References**

Ambekar Institute for Labour Studies (2009) *Living Wage Survey for India - The Report*. Available at: <http://www.fes.or.id/fes/download/Survey_Result_India.pdf> (Accessed: 18/11/2015.

Anker, R. (2006) 'Living wages around the world: A new methodology and internationally comparable estimates', *International Labour Review,* 145(4), pp. 309-338.

Anker, R. (2011a) *Engel’s Law Around the World 150 Years Later, PERI Working Paper No. 247*. Available at: <http://core.ac.uk/download/pdf/6307249.pdf> (Accessed: 09/11/2015].

Anker, R. (2011b) *Estimating a living wage: A methodological review.* ILO.

Fairwear Foundation (2009) *study on wages in China*. Available at: <http://www.fairwear.org/ul/cms/fck-uploaded/archive/2010-08/fwf_-_study_on_wages_in_china_en_-_2010.pdf> (Accessed: 29/02/2015.

Merk, J. (2009) *Stitching a decent wage across borders: the Asia floor wage proposal*. Globalization & the Workplace 429. Available at: <http://digitalcommons.ilr.cornell.edu/cgi/viewcontent.cgi?article=1423&context=globaldocs> (Accessed: 01/02/2015.

Murgai, R. and Ravallion, M. (2005) 'Employment Guarantee in Rural India: What Would It Cost and How Much Would It Reduce Poverty?', *Economic and Political Weekly,* 40(31), pp. 3450-3455

Rani, U. and Belser, P. (2012) 'Low pay among wage earners and the self-employed in India', *International Labour Review,* 151(3), pp. 221-242.

Xu, Z., Chen, Y. and Li, M. (2015) 'Are Chinese Workers Paid the Correct Wages? Measuring Wage Underpayment in the Chinese Industrial Sector, 2005-2010', *Review of Radical Political Economics,* 47(3), pp. 446-459.
