# Supplementary material for: Investigating fairness in global supply chains: applying an extension of the living wage to the Western European clothing supply chain
Source: Int J Life Cycle Assess. 2017 Aug 30;23(9):1862–73. doi: 10.1007/s11367-017-1390-z (PMC6428394; doi:10.1007/s11367-017-1390-z)
Supplement: Supplementary file 4 — (DOCX 25 kb) [file 11367_2017_1390_MOESM4_ESM.docx]

**Appendix D**

Table D1 Sector classification system correspondence table

| **Chapter 3, of this Thesis** | **WIOD** | **NACE Codes** |
| --- | --- | --- |
| Agriculture | Agriculture | AtB |
| Energy and Resources | Mining and Quarrying, Coke; Refined Petroleum and Nuclear Fuel; Electricity, Gas and Water Supply | C;23;E |
| Textiles and Clothing | Textiles and Clothing | 17t18 |
| Other Manufactures | Food, Beverages and Tobacco; Leather, Leather and Footwear; Wood and Products of Wood and Cork; Pulp, Paper, Paper , Printing and Publishing; Chemicals and Chemical Products; Rubber and Plastics; Other Non-Metallic Mineral; Basic Metals and Fabricated Metal; Machinery, Nec; Electrical and Optical Equipment; Transport Equipment; Manufacturing, Nec; Recycling; Construction; Sale, Maintenance and Repair of Motor Vehicles and Motorcycles; Retail Sale of Fuel; Wholesale Trade and Commission Trade, Except of Motor Vehicles and Motorcycles; Retail Trade, Except of Motor Vehicles and Motorcycles; Repair of Household Goods | 15t16; 19;20; 21t22; 24; 25; 26; 27t28; 29; 30t33; 34t35 36t37;F;50; 51; 52 |
| Services | Hotels and Restaurants; Inland Transport; Water Transport; Air Transport; Other Supporting and Auxiliary Transport Activities; Activities of Travel Agencies; Post and Telecommunications; Financial Intermediation; Real Estate Activities; Renting of M&Eq and Other Business Activities; Public Admin and Defence; Compulsory Social Security; Education; Health and Social Work; Other Community, Social and Personal Services; Private Households with Employed Persons | H; 60; 61; 62; 63; 64; J; 70; 71t74; L; M; N; O; P |
